# P-799. Eravacycline in the Management of Nontuberculous Mycobacteria (NTM) Infections: A Single-Centre Experience

**DOI:** 10.1093/ofid/ofae631.991

**Published:** 2025-01-29

**Authors:** Narendran Koomanan, Peijun Yvonne Zhou, Lay Hoon Andrea Kwa, Shimin Jasmine Chung

**Affiliations:** Singapore General Hospital, Singapore, Not Applicable, Singapore; Singapore General Hospital, Singapore, Not Applicable, Singapore; Singapore General Hospital, Singapore, Not Applicable, Singapore; Singapore General Hospital, Singapore, Not Applicable, Singapore

## Abstract

**Background:**

Ubiquitous in nature, nontuberculous mycobacteria (NTM) have been associated with a wide spectrum of clinical infections. Treatment often requires the use of multiple antibiotics for a prolonged course. However, due to inherent drug resistance & treatment-limiting adverse events (AE), clinical outcomes are often suboptimal. In vitro studies have suggested that eravacycline (ERV) might be an effective component of NTM treatment regimens. However, clinical studies showing its safety & efficacy are limited. Hence, this study aims to assess the safety & efficacy of ERV treatment in NTM infections.

**Table 1:**
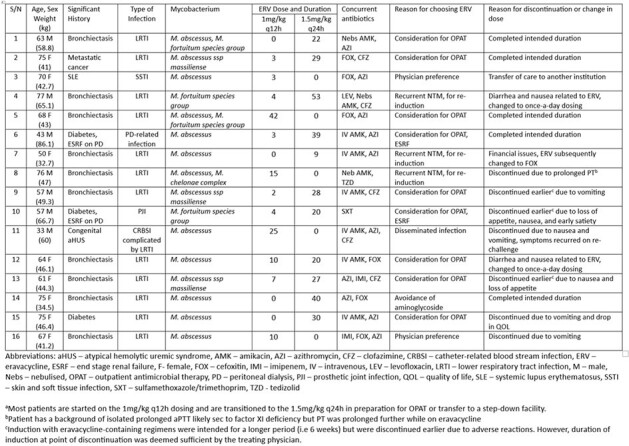
Clinical Characteristics of Patients Receiving Eravacycline (ERV) for the Treatment of NTM Infections

**Methods:**

A retrospective, single-centre observation study reviewing the electronic medical records of all patients prescribed at least 48 hours of ERV from May 22 to April 23 for the treatment of NTM infections was conducted. For each course of ERV, demographic, microbiological & clinical outcome data was collected. Efficacy outcomes included treatment outcome at end of therapy. Safety outcomes included presence of any AE that led to discontinuation of ERV.

**Results:**

During the study period, 16 patients received ERV containing regimens for the treatment of NTM infections (Table 1). A majority (75%) were being treated for pulmonary NTM infections & *M. abscessus* was the most isolated mycobacterium (87.5%). Treatment consisted of ERV, in combination with 2-3 antibiotics, for a median duration of 30 days (IQR: 20.3-35.5 days). To facilitate outpatient antimicrobial therapy (OPAT), most patients were transitioned from 1mg/kg q12h to 1.5mg/kg q24h dosing, with half (50%) eventually completing treatments via OPAT. At the end of ERV therapy, all patients demonstrated clinical improvement. AE led to discontinuation of ERV in 7 (43.8%) patients & dose reduction in 2 (12.5%) patients. Gastrointestinal (GI) AE was most prevalent (8/9, 88.9%) with improvements observed in both patients with dose reductions. Despite the truncated therapy, clinical improvement was still observed amongst patients with AE.

**Conclusion:**

ERV would bolster the armamentarium of antibiotics against NTM infections, especially with its OPAT compatibility. However, in our population, a considerable proportion could not tolerate ERV. Dose reduction maybe be considered to improve ERV tolerability.

**Disclosures:**

**All Authors**: No reported disclosures

